# Meat Mutagens and Colorectal Adenoma and Cancer: A Problem with a Recently Published Systematic Review and Meta-Analysis

**DOI:** 10.3390/nu10030312

**Published:** 2018-03-06

**Authors:** Ngoan Tran Le

**Affiliations:** 1 Department of Occupational Health, Hanoi Medical University, 1st Ton That Tung street, Dong Da, Hanoi 100000, Vietnam; letngoan@hmu.edu.vn or lengoan@iuhw.ac.jp; Tel.: +84-24-3852-3798 or +81-476-20-7701; 2 Department of Public Health, School of Medicine, International University of Health and Welfare, 4-2 Kozunomori, Narita, Chiba 286-8686, Japan

Dear Editors and Authors,

I had the pleasure to read the published article entitled [1] “Dietary Intake of Meat Cooking-Related Mutagens (HCAs) and Risk of Colorectal Adenoma and Cancer: A Systematic Review and Meta-Analysis”. The authors completed a thorough collection and examination of thirty nine studies in their systematic review and meta-analysis. The aim of the work was to examine the association between heterocyclic amines (HCAs) intake and the risk of colorectal cancer (CRC) and colorectal adenoma (CRA) through a systematic review and meta-analysis. HCAs included 2-amino-1-methyl-6-phenylimidazo[4,5-*b*]pyridine (PhIP), 2-amino-3,8-dimethylimidazo[4,5-*f*]quinoxaline (MeIQx) and 2-amino-3,4,8-trimethylimidazo[4,5-*f*]quinoxaline (DiMeIQx) and the “meat-derived mutagenic activity” (MDM).

This work is very necessary and useful for engendering discussion regarding risk factors related to cancer.

The main findings included “Polled CRA risk (15,229 cases) was significantly increased by intake of PhIP (OR (odds ratio) = 1.20; 95% CI (confidence interval): 1.13, 1.28; *p* < 0.001), MeIQx (OR = 1.14; 95% CI: 1.05, 1.23; *p* = 0.001), DiMeIQx (OR = 1.13; 95% CI: 1.05, 1.21; *p* = 0.001), B(a)P (benzo(a)pyrene) (OR = 1.10; 95% CI: 1.02, 1.19; *p* = 0.017) and MDM (OR = 1.17; 95% CI: 1.07, 1.28; *p* = 0.001). A linear and curvilinear trend was observed in dose–response meta-analysis between CRA risk in association with PhIP, MDM, and MeIQx. “CRC risk (21,344 cases) was increased by uptake of MeIQx (OR = 1.14; 95% CI: 1.04, 1.25; *p* = 0.004), DiMeIQx (OR = 1.12; 95% CI: 1.02, 1.22; *p* = 0.014) and MDM (OR = 1.12; 95% CI: 1.06, 1.19; *p* < 0.001). No publication bias could be detected, whereas heterogeneity was in some cases rather high. Mutagenic compounds formed during cooking of meat at high temperature may be responsible of its carcinogenicity” (Abstract).

However, upon careful review of the number of data used to calculate risk in the study, some findings from the meta-analysis appear perplexing. This may result from a substantial problem in repeated usage of data; specifically, possible repeated examinations of OR/RR (relative risk) (95% CI), and missing data in prospective cohort studies. These issues might result in wrong outputs and, thus, skewed findings of the present study.

## 1. Combined Data from Prospective Cohort Study and Case-Control Study

It appears the authors’ above conclusion did not separate the pooled analysis in each study design within the Prospective Cohort Study and Case-Control Study. Those designs definitely differed regarding exposure measures of HCA intake before the occurrence of colorectal adenoma and cancer (Prospective Cohort Study) and after these diseases occurred (Case-Control Study). The findings presented (in Table 1 of the published article) [1] for the pooled analysis of Case-Control Studies indicated that there is a significant positive association between HCA intake and the risk of colorectal adenoma for all PhIP, MeIQx, DiMeIQx, and MDM. However, the pooled analysis of the Prospective Cohort Studies has confirmed only PhIP and its significant positive association. 

## 2. Repeated Examination and Possible Partly Repeated Usage Data

### 2.1. For Colorectal Adenoma

Four published articles used participants recruited from 2003 to 2010 from the Tennessee Colorectal Polyp Study [2,3,4,5], a case-control study conducted in Nashville, Tennessee. The most recent cases of colorectal adenoma were 1527 and controls were 3329 cases in 2012. Due to repeated examination and a series of four published papers, the authors cited the total of colorectal adenoma cases as 4538 and controls as 10,130 in the present meta-analysis (Table 1). It appears that the number of colorectal adenoma cases and controls may have been repeatedly examined for HCAs and consequently, input four times in the present Meta-Analysis [1].

### 2.2. For Colorectal Cancer

Two published articles using data from 1991 to 2002 from Utah and Northern California [6,7] for about 2298 cases of colorectal cancer and 2749 controls, of which there were 952 cases of colorectal cancer and 1205 controls recruited from 1997 to 2002 were published in 2004.

Furthermore, three published articles have also used cases (620, published in 2003) and controls (1038, published in 2003) from the North Carolina Colon Cancer Study [8,9,10] from 1996 to 2000 that were all input in the present Meta-Analysis. The present meta-analysis lists the totals from the five published articles including 4777 cases of colorectal cancer and 6253 controls, of which the number of cases and controls might have been repeated two or three times.

Were the findings of the meta-analysis modified due to repeatedly inputting data twice, three or four times? The authors should correct and fix these problems to show accurate results. 

## 3. Possible Repeated Examinations of OR/RR (95% CI)

### 3.1. For Colorectal Adenoma

#### 3.1.1. Case-Control Study

For the study by Sinha, et al. in 2005 [11], the authors used twice OR (95% CI) for both CRC and their sub sites of colon and rectum for both PhIP and MeIQx, Table 2, DiMeIQx and MDM (data not shown). 

#### 3.1.2. Prospective Cohort Study

For the study by Rohrman, et al. in 2009 [12], the authors used twice OR (95% CI) for both CRC and their sub sites of colon and rectum for PhIP (Table 2).

For the study by Ferrucci, et al. in 2012 [13], the authors used twice OR (95% CI) for both CRC and their sub sites of colon and rectum for both PhIP and MeIQx (Table 2) DiMeIQx and MDM (data not shown). 

By repeated usage of RR (95% CI), there were four published articles of prospective cohort studies only; the number of data points used to calculate the risk were eight for PhIP, six for MeIQx, six for DiMeIQx, and five for MDM [1].

### 3.2. For Colorectal Cancer

#### 3.2.1. Case-Control Study

For the two studies by Miller, et al. in 2013 [14] and Joshi, et al. in 2015 [15], the authors again used twice OR (95% CI) for both CRC and their sub sites of colon and rectum for both PhIP and MeIQx (Table 2) and DiMeIQx (data not shown). For MDM, the doubled OR (95% CI) was seen in the study by Miller, et al. in 2013 [14].

#### 3.2.2. Prospective Cohort Study

For the study by Cross, et al. in 2010 [16], the authors used twice RR (95% CI) for both CRC and their sub sites of colon and rectum for both PhIP and MeIQx (Table 2) DiMeIQx and MDM (data not shown). 

After the doubled RR(95% CI), there were only three published articles of prospective cohort studies, and the number of data points used to calculate the risk were five for PhIP, MeIQx, and DiMeIQx, and four for MDM [1].

Among the available three prospective cohort studies, only the study by Cross, et al. in 2010 [16] showed a significant positive association between HCAs intake and the risk of CRC for MeIQx, DiMeIQx and MDM. Due to the repeated usage of those RR (95% CI), the findings of the present meta-analysis might result in an over-positive estimation of the pooled analysis of prospective cohort studies. 

Again, were the findings of the meta-analysis modified due to double input of OR/RR (95% CI) data? The authors should correct and fix these problems to show accurate results.

## 4. Missing Data from Prospective Cohort Studies

The data of MDM was missing from the study by Ollberding, et al. in 2012 [17]. The pointed estimation of RR was 1.01 for MeIQx, but less than one (negative association) for PhIP (0.95), for DiMeIQx (0.88) and total HCAs (0.90). Due to missing MDM (Ollberding, et al. in 2012) and doubled RR (95% CI): 1.14 (1.01, 1.29) (Cross, et al. in 2010 [16], significant positive association) (Table 3). The estimated risk of CRC was RR (95% CI): 1.12 (1.03, 1.21), *p* Value = 0.005 that might not reflect the true findings of the pooled analysis of only two available studies of prospective cohort studies. 

I address these points only to illustrate the subtleties in handling data used to calculate risk and the importance of avoiding repeated usage of cases and controls due to multiple publications. For example, for OR/RR (95% CI), if analysis includes CRC, then the usage of their sub sites of colon and rectum in the computer analyzing programs will result in errant data in a meta-analysis. The authors should rerun the meta-analysis after excluding repeated cases and controls and avoid double OR/RR (95% CI). I believe this will result in accurate outputs and findings.

## Figures and Tables

**Table 1:** Repeated examination and possible repeated data.

Study (Year)	Time Recruited Cases and Controls	Cases	Controls	Journal	Possible Repeated Data
**Colorectal adenoma**					
Fu (2012) [2]	2003–2010	1527	3329	Am. J. Clin. Nutr.	Four times
Fu (2011) [3]	2003–2010	1881	3764	Cancer Prev. Res.	
Shin (2008) [4]	2003–2005	557	1493	Cancer Epi. Bio. and Prevention	
Shin (2007) [5]	2003–2005	573	1544	Int. J. Cancer	
**Total colorectal adenoma**		**4538**	**10,130**		
**Colorectal cancer**					
Murtaugh (2004) [6]	1997–2002	952	1205	Journal of nutrition	Two times
Murtaugh (2005) [7]	1991–2002	2298	2749	Journal of nutrition	
Butler (2003) [8]	1996–2000	620	1038	American Journal of Epidemiology	Three times
Butler (2005) [9]	1996–2000	400	412	Cancer Epi. Bio. & Prevention	
Butler (2008) [10]	1996–2000	507	849	Mutation research	
**Total Colorectal cancer**		**4777**	**6253**		

**Table 2:** Possible repeated examinations of OR/RR (95% CI).

Study (Year)	Cases	Controls	PhIP	MeIQx
			OR/RR (95% CI)	OR/RR (95% CI)
Colorectal adenoma				
Case-control study				
Sinha (2005) [11]	3696	34,817	Colon: 1.17 (1.01–1.35)	Colon: 1.18 (1.01–1.38)
			Rectal: 1.02 (0.79–1.33)	Rectal: 0.79 (0.60–1.04)
			CRC: 1.11 (0.98–1.25)	CRC: 1.08 (0.95–1.23)
Prospective cohort studies	Cases	Participants		
Rohrmann (2009) [12]	516	25,540	Colon: 1.56 (1.12–2.19)	-
			Rectal: 1.08 (0.62–1.86)	-
			CRC: 1.47 (1.13–1.93)	-
Ferrucci (2012) [13]	1008	17,072	Colon: 1.07 (0.85–1.36)	Colon: 0.97 (0.76–1.24)
			Rectal: 1.75 (1.17–2.64)	Rectal: 1.12 (0.74–1.72)
			CRC: 1.18 (0.96–1.45)	CRC: 0.99 (0.80–1.23)
**Total (Prospective cohort studies)**	**1524**	**42,612**		
Colorectal cancer				
Case-control study	Cases	Controls		
Miller (2013) [14]	989	1033	Colon: 0.95 (0.68–1.33)	Colon: 1.23 (0.89–1.69)
			Rectum: 1.33 (0.88–2.02)	Rectum: 1.24 (0.81–1.91)
			CRC: 1.06 (0.79–1.43)	CRC: 1.22 (0.91–1.64)
Joshi (2015) [15]	3350	3504	Colon: 1.00 (0.80–1.20)	Colon: 1.10 (0.90–1.30)
			Rectum: 0.90 (0.70–1.10)	Rectum: 0.90 (0.70–1.20)
			CRC: 0.90 (0.80–1.10)	CRC: 1.00 (0.90–1.20)
**Total case-control**	**4339**	**4537**		
Prospective cohort studies	Cases	Participants		
Cross (2010) [16]	2719	300,948	Colon: 1.01 (0.87–1.16)	Colon: 1.26 (1.09–1.45)
			Rectum: 0.94 (0.73–1.20)	Rectum: 1.01 (0.79–1.28)
			CRC: 0.99 (0.87–1.12)	CRC: 1.19 (1.05–1.34)

PhIP: 2-amino-1-methyl-6-phenylimidazo[4,5-*b*]pyridine, MeIQx: 2-amino-3,8-dimethylimidazo[4,5-*f*]quinoxaline, OR: odds ratio, RR: relative risk, CI: confidence interval, CRC: colorectal cancer.

**Table 3:** Missing data from prospective cohort studies.

Exposure Indicator of HCAs	Le (2016) * [18]	Ollberding (2012) * [17]	Cross (2010) * [16]
PhIP	CRC: 1.09 (0.90–1.33)	CRC: 0.95 (0.81–1.11)	CRC: 0.99 (0.87–1.12)
MeIQx	CRC: 1.12 (0.93–1.34)	CRC: 1.01 (0.86–1.19)	CRC: 1.19 (1.05–1.34)
DiMeIQx	CRC: 1.05 (0.88–1.25)	CRC: 0.88 (0.75–1.03)	CRC: 1.17 (1.05–1.29)
Total HCAs	-	CRC: 0.90 (0.76–1.05)	-
MDM	CRC: 1.03 (0.86–1.24)	-	CRC: 1.14 (1.01–1.29)

HCAs: heterocyclic amines, PhIP: 2-amino-1-methyl-6-phenylimidazo[4,5-*b*]pyridine, MeIQx: 2-amino-3,8-dimethylimidazo[4,5-*f*]quinoxaline, DiMeIQx: 2-amino-3,4,8-trimethylimidazo[4,5-*f*]quinoxaline, MDM: meat-derived mutagenic activity, * Study (year), CRC: colorectal cancer.
